# Pseudoexons provide a mechanism for allele-specific expression of *APC* in familial adenomatous polyposis

**DOI:** 10.18632/oncotarget.12206

**Published:** 2016-09-23

**Authors:** Taina T. Nieminen, Walter Pavicic, Noora Porkka, Matti Kankainen, Heikki J. Järvinen, Anna Lepistö, Päivi Peltomäki

**Affiliations:** ^1^ University of Helsinki, Medical and Clinical Genetics, Helsinki, Finland; ^2^ Laboratorio de Citogenética y Mutagénesis, Instituto Multidisciplinario de Biología Celular (IMBICE-CONICET-CICPBA), La Plata, Argentina; ^3^ University of Helsinki, Institute for Molecular Medicine Finland, Helsinki, Finland; ^4^ Second Department of Surgery, Helsinki University Central Hospital, Helsinki, Finland; ^5^ Department of Colorectal Surgery, Abdominal Center, Helsinki University Hospital, Helsinki, Finland

**Keywords:** familial adenomatous polyposis, APC, pseudoexon, RNA-seq, allele-specific expression

## Abstract

Allele-specific expression (ASE) of the Adenomatous Polyposis Coli (*APC*) gene occurs in up to one-third of families with adenomatous polyposis (FAP) that have screened mutation-negative by conventional techniques. To advance our understanding of the genomic basis of this phenomenon, 54 *APC* mutation-negative families (21 with classical FAP and 33 with attenuated FAP, AFAP) were investigated. We focused on four families with validated ASE and scrutinized these families by sequencing of the blood transcriptomes (RNA-seq) and genomes (WGS). Three families, two with classical FAP and one with AFAP, revealed deep intronic mutations associated with pseudoexons. In all three families, intronic mutations (c.646-1806T>G in intron 6, c.1408+729A>G in intron 11, and c.1408+731C>T in intron 11) created new splice donor sites resulting in the insertion of intronic sequences (of 127 bp, 83 bp, and 83 bp, respectively) in the *APC* transcript. The respective intronic mutations were absent in the remaining polyposis families and the general population. Premature stop of translation as the predicted consequence as well as co-segregation with polyposis supported the pathogenicity of the pseudoexons. We conclude that next generation sequencing on RNA and genomic DNA is an effective strategy to reveal and validate pseudoexons that are regularly missed by traditional screening methods and is worth considering in apparent mutation-negative polyposis families.

## INTRODUCTION

Familial adenomatous polyposis (FAP; OMIM #175100) is characterized by a dominant predisposition to multiple adenomatous polyps throughout the colon and rectum as a consequence of germline mutations in the Adenomatous Polyposis Coli (*APC*) gene [1]. While FAP mostly represents an inherited disease, up to 25% may result from *de novo* mutations of *APC* without any family history of the disease [2]. The number of adenomatous polyps in the bowel is used to stratify *APC*-associated polyposis into a classical form (FAP; 100 adenomas or more) and attenuated form (AFAP; below 100 adenomas). These two phenotypes additionally differ relative to the onset of polyposis (in the second or third decades of life in FAP vs. later in AFAP), colonic location (left-sided disease in FAP vs. frequently right-sided disease in AFAP), and life-time risk of colorectal cancer (100% in FAP vs. up to 70% in AFAP) [1, 3].

The *APC* gene has 16 exons and translation starts from exon 2 (http://insight-database.org/genes/APC). More than 1,500 unique germline mutations in *APC* are known [4]. The frequency of detectable *APC* mutations in polyposis patients varies a lot depending on the method of ascertainment of the patients and families, and the strategies used for mutation screening. In a large cohort of individuals who had undergone clinical genetic testing because of a personal or family history of polyposis, 58% (851/1457) of those with classic polyposis and 9% (376/4223) of those with AFAP had *APC* mutations by exon-specific sequencing and large rearrangement analysis of the *APC* gene [3]. Moreover, Grover et al. [3] found that the *APC* mutation rate progressively increased with the cumulative adenoma count (being 80% in individuals with at least one thousand adenomas), while the mutation rate of *MUTYH*, which is another polyposis-associated gene, remained constant (below 10 percent) across all polyp number categories. Sanger sequencing of genomic DNA to examine the coding exons and intron-exon boundaries of *APC*, combined with multiplex ligation-dependent probe amplification (MLPA) for large rearrangements is the standard mutation screening strategy adopted by most laboratories [4]. The protein truncating test (PTT) was commonly used in previous years and may be beneficial in certain situations [5]. In a typical PTT design, *APC* exons are examined in RNA, except for the last exon that is investigated in genomic DNA. Nevertheless, over 20% of classical FAP and up to 80% of AFAP patients remain *APC* mutation-negative, which may be attributable to methodological shortcomings in association with particular types of mutations [5–8], nontruncating alterations with uncertain pathogenic significance [2], and susceptibility associated with other genes than *APC*, such as *MUTYH* [3], *POLE* and *POLD* [9], and *AXIN2* [10].

Unbalanced expression of the two parental alleles, due to loss-of-function mutations or various *cis-* or *trans*-acting factors, may facilitate the identification of susceptibility genes for human diseases [11]. *APC* mutations occurring prior to the last exon of the gene are associated with allele-specific expression (ASE) [12]. ASE imbalance of *APC* has been found in blood samples from 9 – 31% of adenomatous polyposis families without any detectable *APC* mutations by conventional techniques, suggesting the existence of hidden mutations [12–14]. Moreover, ASE of *APC* may contribute to common forms of colorectal cancer, as colorectal cancer risk has been shown to increase along with increasing ASE imbalance [15].

This study was undertaken to address the underlying basis of predisposition in 54 *APC* mutation-negative adenomatous polyposis families from Finland, with a particular focus on families with constitutionally unbalanced mRNA expression of *APC* alleles by Single Nucleotide Primer Extension (SNuPE) [13]. Interrogation of the latter four families by whole transcriptome (RNA-seq) and whole-genome (WGS) sequencing revealed deep intronic mutations associated with pseudoexons in three of four families.

## RESULTS

### Identification of pseudoexons by RNA-seq and deep intronic mutations as their underlying causes

**Figure 1:**
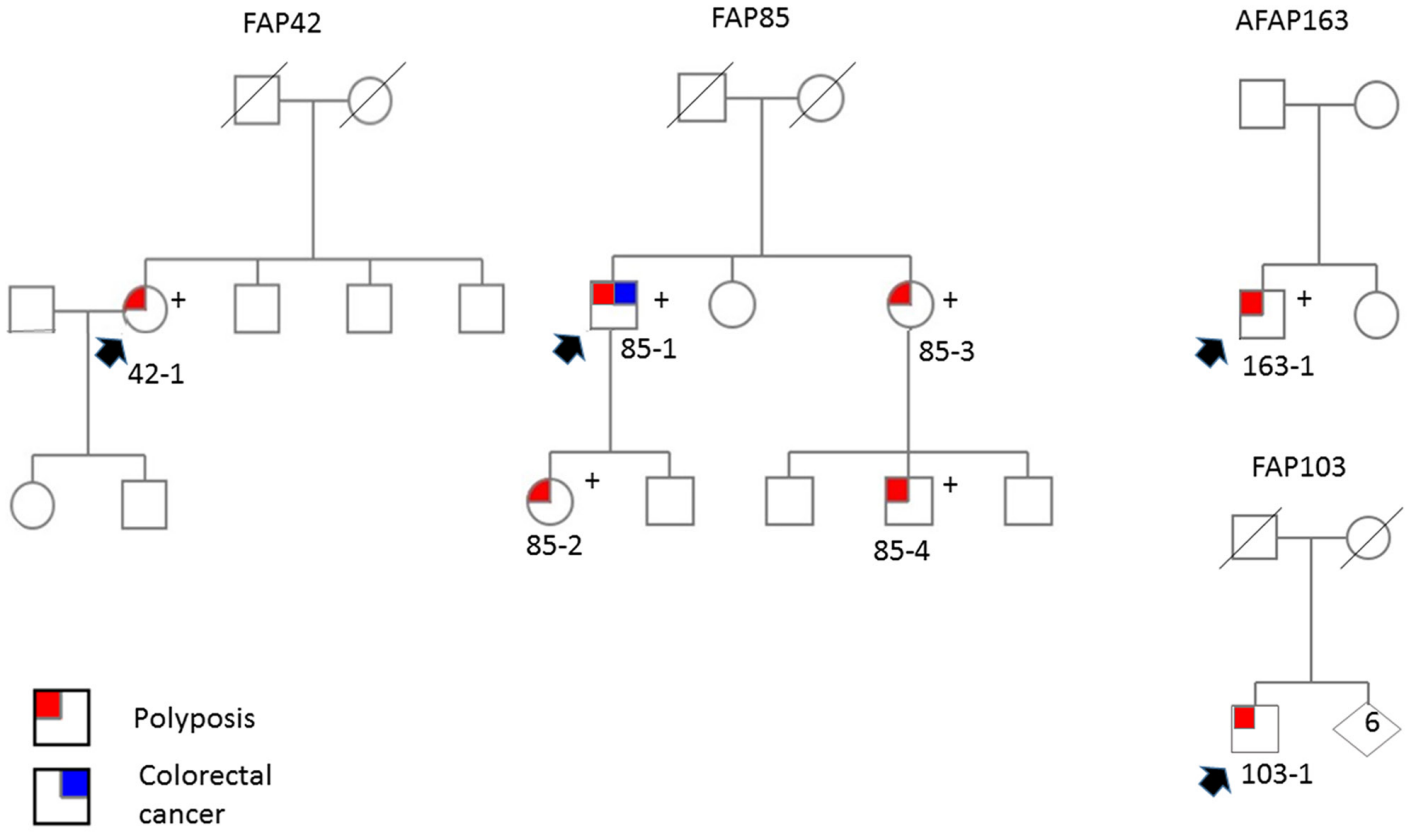
Pedigrees of ASE families Pedigrees of adenomatous polyposis families with ASE. Individuals with polyposis and/or colorectal cancer are indicated (see Table 1 for additional clinical details). Plus sign denotes carriers of deep intronic mutations associated with pseudoexons of *APC*. Index persons are marked with arrows.

We focused on three FAP families (42, 85, and 103) from the research-based cohort (Figure 1 and Table 1). The families were associated with ASE imbalance of *APC* by SNuPE but no identifiable causative change in *APC* had been detected by PTT, Sanger sequencing of all exons and intron/exon borders, MLPA, and promoter mutation and methylation analyses (ref. [8] and this study). Only family 85 included several affected members. Of these, 85-1 [13] and 85-2 (Supplementary Figure S1) showed ASE imbalance, whereas 85-3 was uninformative in ASE analysis due to homozygosity for polymorphisms. No RNA was available from 85-4.

**Table 1:** Clinical and molecular characteristics of polyposis cases investigated

	Case ID^a^	ASE status^b^	Inheritance pattern	Number of polyps	Age at diagnosis^c^	Extracolonic manifestations^d^	Classification of family^e^	Large rearrangement by MLPA^f^	*APC* methylation by MS-MLPA^g^
RESEARCH BASED	42	ASE	Sporadic	100-1000	40	No	FAP	No	No
	78	N	Sporadic	50	55	No	AFAP	No	No
	85-1	ASE	dominant	2000	38	Yes	FAP	No	No
	85-2	ASE	dominant	100-200	16	No	FAP	No	No
	85-3	NI	dominant	2000	44	No	FAP	NA	No
	85-4		dominant	100-200	12	No	FAP	NA	No
	88	N	sporadic	100-1000	58	No	FAP	No	No
	92	N	sporadic	200	51	No	FAP	No	No
	96	NI	sporadic	561	48	No	FAP	No	No
	97	N	sporadic	> 1000	58	No	FAP	No	No
	98		dominant	100-1000	30	No	FAP	No	No
	100	NI	sporadic	30	62	No	AFAP	No	No
	103	(ASE)	sporadic	> 100	51	No	FAP	No	No
	104	N	dominant?	210	54	Yes	FAP	No	No
	111	NI	sporadic	30-40	36	No	AFAP	No	No
	123	NI	sporadic	2100	37	No	FAP	No	No
	125	N	sporadic	300	31	No	FAP	No	No
CLINIC BASED	134		sporadic	200-300	55	No	FAP	No	No
	136		sporadic	>100	67	Yes	FAP	No	No
	139		sporadic	100	71	No	FAP	No	No
	145		recessive	20-50	61	No	AFAP	No	No
	148		sporadic	150-200	50	No	FAP	No	No
	158		sporadic	50	49	Yes	AFAP	No	No
	159		sporadic	200	50	No	FAP	No	No
	162		sporadic	>50	52	No	AFAP	No	No
	163	(ASE)	sporadic	10-20	16	Yes	AFAP	No	No
	165-1		dominant?	Colon cancer x 2	50	NA	AFAP	No	No
	165-2		dominant?	20-30	33	Yes	AFAP	No	No
	168		sporadic	100	56	Yes	FAP	No	No
	177		sporadic	100-200	52	No	FAP	No	No
	179		dominant?	>10	23	No	AFAP	No	No
	180		NA	>100	38	NA	FAP	No	No
	1001		dominant	10	48	NA	AFAP	No	No
	1003		sporadic	20-30	70	NA	AFAP	No	No
	1005		dominant	10-20	68	Yes	AFAP	No	No
	1006		sporadic	20	60	No	AFAP	No	No
	1007		sporadic	20	30	No	AFAP	No	No
	1010		dominant	5-10	68	NA	AFAP	No	No
	1011		NA	60-100	31	NA	FAP	No	No
	1013		sporadic	>100	48	NA	FAP	No	No
	1015		sporadic	10	47	Yes	AFAP	No	No
	1017		sporadic?	10-20	57	NA	AFAP	No	No
	1018		sporadic	20-30	74	NA	AFAP	No	No
	1019		sporadic	2-3	30	Yes	AFAP	No	No
	1020		sporadic	3	35	Yes	AFAP	No	No
	1021		sporadic	30	72	NA	AFAP	No	No
	1022		dominant	3	65	Yes	AFAP	No	No
	1023		sporadic	40	33	NA	AFAP	No	No
	1024		sporadic	20	72	NA	AFAP	No	No
	1025		sporadic	20-30	67	NA	AFAP	No	No
	1026		sporadic	10-20	51	NA	AFAP	No	No
	1029		sporadic	20-30	56	No	AFAP	No	No
	1030		sporadic	>10	59	No	AFAP	No	No
	1032		NA	8	63	Yes	AFAP	No	No
	1034		NA	>10	62	No	AFAP	No	No
	1035		sporadic	20-30	71	No	AFAP	No	No
	1036		dominant	>10	61	No	AFAP	No	No
	1037		sporadic	~10	52	No	AFAP	No	No

a Identification number of family, followed by identification number of individual if several family members were studied.

b ASE, shows allele-specific expression of *APC;* (ASE), putative ASE (see Materials and Methods); N, no ASE; NI, not informative (homozygous); blank, no RNA available

c Polyposis or colorectal carcinoma, whichever comes first

d Desmoids and duodenal adenomas in particular

e Based on the number of intestinal adenomas with 100 as the cut-off

f P043-C1 assay from MRC-Holland

g ME001-C1 assay from MRC-Holland

NA, information not available

**Figure 2:**
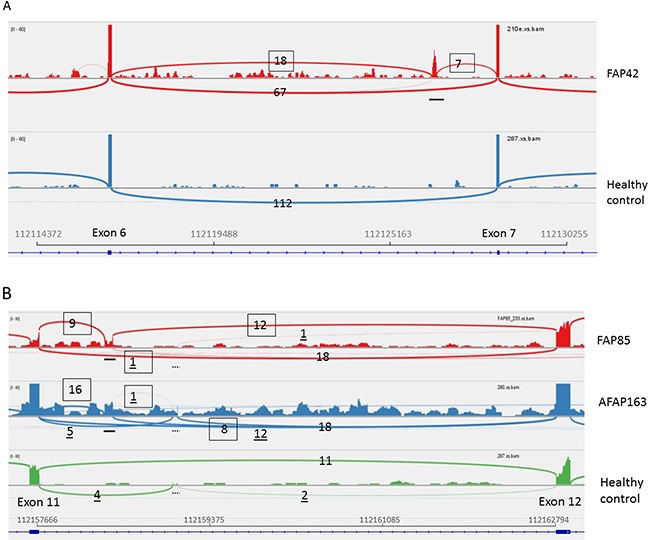
RNA-seq (42, 85-2, and 163) Sashimi plots to visualize splice junctions. IGV display of RNA-seq data is provided for an affected representative of each family and a healthy control individual for reference for each region. Sequence alignments are based on TopHat. The region between *APC* exons 6 and 7 (GRCh37/Hg19) is shown for FAP42 (Figure 2A) and that between exons 11 and 12 for FAP85 (individual 85-2) and AFAP163 (Figure 2B). The locations of pseudoexons are indicated by horizontal bars. A 54-bp in-frame insertion present in the normal reference sample, too, and not associated with any genomic change is denoted by a dashed bar (Figure 2B). The same insertion was discovered in an earlier investigation [12]. Numbers on the plots indicate *APC* exon coverages expressed as junction depth. Splice events corresponding to pseudoexons are boxed and those associated with the 54-bp insertion are underlined; the remaining ones represent canonical splicing.

**Figure 3:**
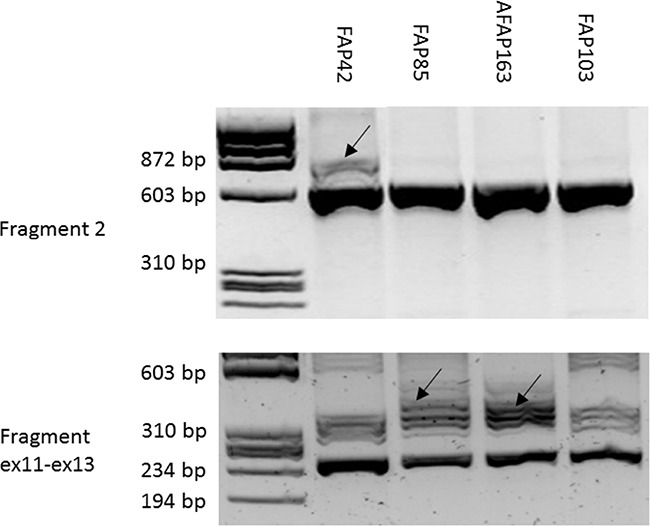
RT-PCR (42, 85-1, 163, 103) RT-PCR analysis of samples from ASE families. RT-PCR products separated by gel electrophoresis are shown. Arrows denote fragments with intronic insertions (pseudoexons). Fragment 2 (upper panel) encompasses a 615-bp cDNA segment from exon 4 to exon 9 [6] and shows a heterozygous 127-bp insertion in family 42. The wild-type size of the exon 11 - exon 13 fragment (lower panel) is 246 bp (Supplementary Table S1). An identical 83-bp insertion in families 85 (case 85-1) and 163 is evident. The RT-PCR products from the index persons and healthy controls were cloned and sequenced to verify their DNA sequences. In the exon 11 - exon 13 fragment, a 54-bp in-frame insertion (see legend for Figure 2) accompanied the pseudoexon and wild-type sequences in a proportion (up to one-third) of all clones and likely contributed to the multiplicity of fragments seen after gel electrophoresis.

Blood RNA specimens from the three ASE families were subjected to RNA-seq. Data analysis revealed aberrant splice junctions which raised a suspicion of pseudoexons, i.e., inclusion of intronic sequence in the mature mRNA, in families 42 and 85 (Figure 2). To verify pseudoexons, *APC* cDNA was amplified in five overlapping fragments with primers described in Spier et al. [6] in addition to which primers from exons 11 (forward) and 13 (reverse) were used to evaluate the suspected pseudoexon in family 85 (Supplementary Table S1 and Figure 3). Sequencing of reverse transcription (RT)-PCR products (fragment 2 in family 42 and fragment 4 as well as the exon 11-13-specific fragment in family 85) revealed a 127-bp insertion from intron 6 in family 42 and an 83-bp insertion from intron 11 in family 85.

**Figure 4:**
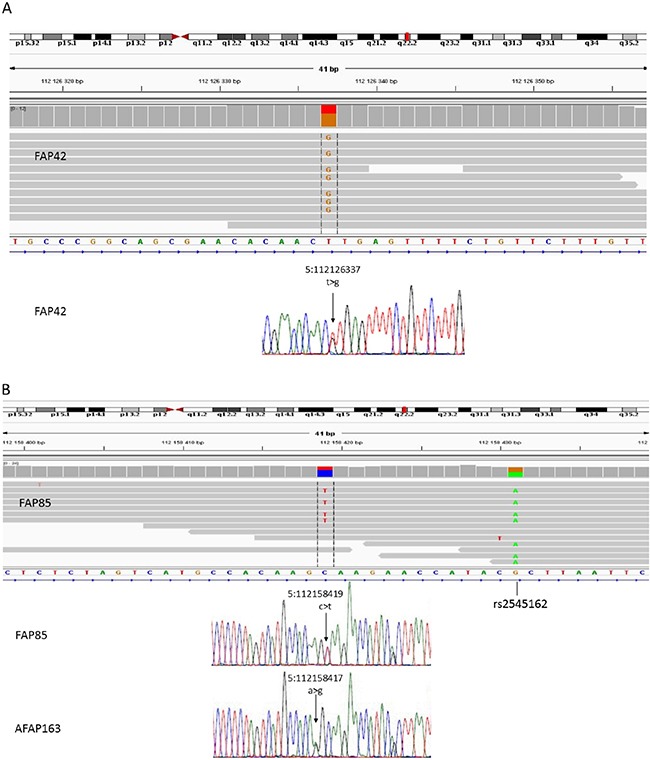
WGS (42 and 85-2) + Sanger seq Deep intronic mutations in *APC*. Upper panels provide IGV display of WGS data for intron 6 in FAP42 (Figure 4A) and intron 11 in FAP85, individual 85-2 (Figure 4B). Lower panels show Sanger sequence tracings of the mutations. In Figure 4B, the Sanger sequencing result of AFAP163 is also given (AFAP163 was not included in WGS analysis).

As the predisposing mutations of the families were unknown, WGS on blood DNA was applied. At the outset, mutations in the *APC* coding region and exon/intron borders had been screened for (see Materials and Methods). Particularly, WGS offered the opportunity to investigate the entire introns of *APC* as well as regions outside *APC*. Families 42 and 85 revealed deep intronic mutations, both creating new splice donor sites (/gt): c.646-1806T>G in intron 6 and c.1408+731C>T in intron 11 of *APC*, respectively (Figure 4). The changes were validated by Sanger sequencing.

**Figure 5:**
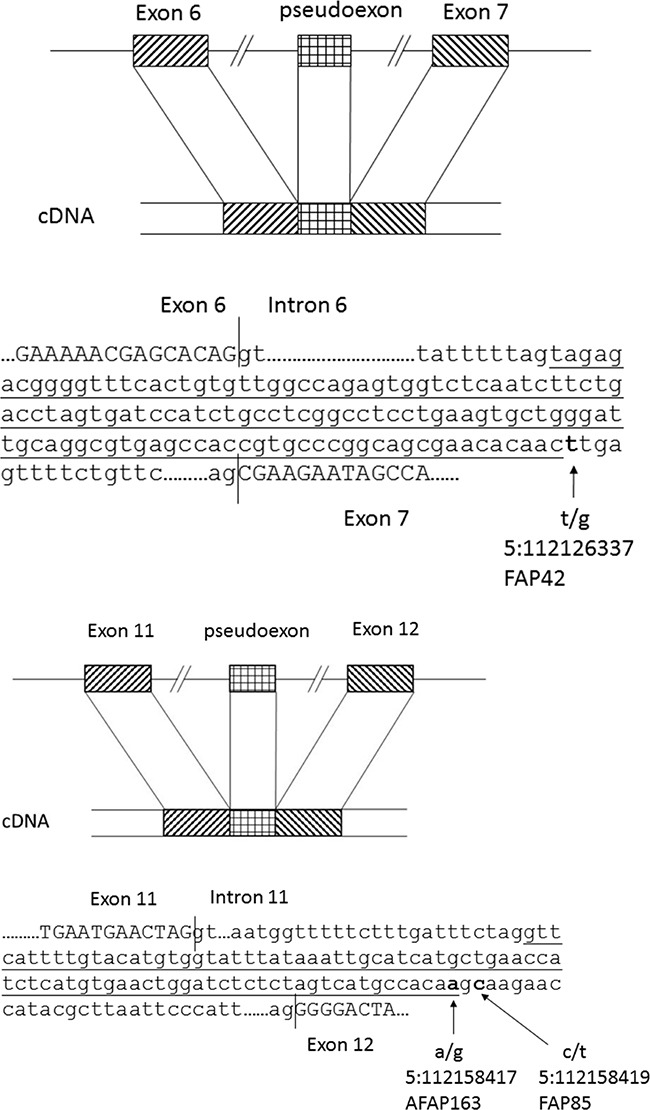
Schematic diagrams of pseudoexons Schematic diagrams of *APC* pseudoexons identified. The canonical splice sites at the exon/intron borders, pseudoexons (underlined), and the responsible deep intronic mutations (in bold) are highlighted.

The remaining 51 families (Table 1) were subsequently screened by Sanger sequencing with primers from introns 6 and 11 of *APC* (Supplementary Table S1) to examine the presence of the deep intronic mutations identified in families 42 and 85. These particular mutations were absent in the remaining families. Incidentally, however, family 163 revealed another nucleotide substitution (c.1408+729A>G) two nucleotides upstream of the mutation present in family 85 (Figure 4). The nucleotide change in family 163 was predicted to activate the same cryptic splice donor site (AG/gt) as the mutation in family 85 (the nucleotide substitutions created an apparently viable AG/ and /gt, respectively) (Figure 5). Family 163 represented a clinic-based cohort for which only DNA was routinely available. However, we were able to obtain RNA from the single affected family member in a separate effort. RNA-seq (Figure 2) and RT-PCR (Figure 3) identified an 83-bp insertion from intron 11, identical to that in family 85. Furthermore, the c.1408+729A>G mutation was part of the resulting transcript unlike the deep intronic mutations of families 42 and 85 (Figure 5). Analysis of the individual RNA reads with pseudoexons validated the presence of variant nucleotide (G) at the position of the mutation, indicating that the variant nucleotide was specifically associated with pseudoexon formation (Supplementary Figure S2). Finally, SNuPE analysis of cDNA from the index individual from family 163 showed putative ASE with the value of 1.7 for the ratio of allelic peak areas in cDNA relative to genomic DNA at rs2229992 (Supplementary Figure S1).

### Pathogenicity of pseudoexons

The pseudoexon findings are summarized in Table 2. The pseudoexons in families 42, 85, and 163 were all predicted to cause premature stop of translation; the very first three nucleotides of the pseudoexon in family 42 coded for a stop of translation, whereas in families 85 and 163 the pseudoexon caused a frameshift and a premature stop 55 codons later. The following evidence supports the idea that the pseudoexons underlay polyposis predisposition in all three families. First, the splice prediction program BDGP (Materials and Methods) indicated a splice efficiency of 99% for the new splice donor sites introduced in intron 6 in family 42 and intron 11 in family 85. The new splice donor site in intron 11 in family 163 did not match with the canonical splice site model and was therefore not recognized by the splice prediction programs. However, RNA-seq and our cloning experiment showed that all pseudoexon-containing transcripts had the variant nucleotide G in the 3' end of the pseudoexon (Supplementary Figure S2). Moreover, our cloning experiment (on the ex 11 – 13 fragment, see legend to Figure 3) combined with haplotype analysis (with SNuPE markers) suggested that all transcripts representing the mutant allele, as inferred from haplotypes, had the pseudoexon inserted (data not shown). Second, the intronic variant showed a complete co-segregation with polyposis in family 85 (Figure 1). The variants were also absent in the general population (ExAC Browser Beta, SISu and Ensembl databases and our investigation of 300 anonymous blood donors from Finland). Finally, WGS data available for families 42 and 85 revealed no other apparently pathogenic mutations in established cancer genes as possible alternative explanations for polyposis predisposition.

**Table 2:** Summary of the variants

Family	Location in *APC* (GRCh37/GRCh38)	Insertion length (bp)	Genomic variant	RNA alteration	Predicted protein alteration
FAP42	intron 6 (5:112126337/5:112790640)	127	c.646-1806T>G	r.645_646ins646-1933_646-1807	p.Arg216*
FAP85	intron 11 (5:112158419/5:112822720)	83	c.1408+731C>T	r.1408_1409ins1408+647_1408+729	p.Gly471Serfs*55
AFAP163	intron 11 (5:112158417/5:112822722)	83	c.1408+729A>G	r.1408_1409ins1408+647_1408+729	p.Gly471Serfs*55

Note: An updated *APC* nomenclature based on 16 exons (NM_012583R138.4, ENST012583R10257430) was used for exon annotation.

### SNuPE vs. RNA-seq in the detection of ASE

The ASE diagnoses of the four families (42, 85, 103, and 163) with unbalanced expression of *APC* alleles in our series (Table 1) were initially based on SNuPE. To evaluate if ASE imbalance was also recoverable in RNA-seq data, a genome-wide ASE imbalance analysis was performed as described in Materials and Methods. The results are given in Supplementary Table S2. Applying stringent criteria for ASE, FAP42 and FAP85 (individual 85-2) revealed unequivocal ASE for *APC* (q-value < 0.05). Three *APC*-mutation-positive cases not belonging to the study series specified in Table 1 were also included, and ASE was detected in one (the remaining two were uninformative). FAP85 (individual 85-1) and FAP103, as well as healthy control sample 3, showed borderline ASE which was, however, not statistically significant after multiple hypothesis correction (q value > 0.05 and ≤ 0.15). The ASE value for *APC* in AFAP163 did not reach statistical significance. As shown in Supplementary Table S2, the overall concordance between ASE results by SNuPE and RNA-seq was high.

## DISCUSSION

Canonical splice-site sequences at the intron/exon borders define exons. The canonical 5' (splice donor) site has a consensus sequence AG/gtragtand the 3' (splice acceptor) site poly(y)nyag/G (where capital letters indicate exonic and lowercase letters intronic sequence, r denotes purine, y pyrimidine, and n any nucleotide, and the nearly invariant nucleotides are underlined) [16]. Pseudoexons are intronic sequences of 50 – 300 bp in length that have apparent 5' and 3' splice sites, but are normally ignored by the splicing machinery [17, 18]. Pseudoexons can be activated by mutations that create viable splice donor or acceptor sites by different mechanisms, resulting in the insertion of intronic sequences in the mature mRNA [19]. Such mutations can be inherited and may cause predisposition to cancer syndromes, including ataxia-telangiectasia (*ATM*) [20], breast and ovarian cancer (*BRCA2*) [21], Lynch syndrome (*MSH2*) [22] and familial adenomatous polyposis (*APC*) (ref. [6] and this study). From the therapeutic point of view, location far outside the coding sequence makes deep intronic mutations excellent candidates for correction by antisense oligonucleotides to restore the production of normal protein [20, 21].

Using RNA-seq and WGS, we discovered two different pseudoexons (127-bp insertion from intron 6 and 83-bp insertion from intron 11) caused by three different heterozygous germline mutations in *APC*. To our knowledge, our effort is the first one successfully identifying pseudoexons in *APC* using next-generation sequencing and the second ever to reveal *APC*-related pseudoexons in FAP. The study by Spier et al. [6] was the first report and described two different *APC* pseudoexons (167-bp insertion from intron 5 and 83-bp insertion from intron 11). These pseudoexons were caused by three different heterozygous germline mutations. By RT-PCR screen of *APC* cDNA from 125 *APC*- and *MUTYH* mutation-negative adenomatous polyposis cases from Germany, a frequency of 6.4% (8/125 individuals) was obtained for cases with an identifiable genomic change underlying pseudoexon formation. Interestingly, the pseudoexon in intron 11 occurring in our family 85 in association with c.1408+731C>T nucleotide substitution was on genomic DNA and RNA level precisely the same as that present in two unrelated German patients [6]. The region around position +731 in intron 11 may be prone to pseudoexon formation in general, given the existence of two additional pseudoexon-associated nucleotide substitutions in this region, one located two nucleotides upstream (our study) and another one six nucleotides downstream of position +731 [6]. The overall frequency of *APC* pseudoexons in our series from Finland (3/54 index patients, 5.5%) may be an underestimate since our full pseudoexon screen focused on four index patients with unbalanced expression of *APC* alleles, whereas the remaining index patients (with mainly DNA available only) underwent a targeted screen for the same mutations identified in the former patients.

Diagnostic strategies mostly target coding regions in DNA [4]. Detection of disease-associated pseudoexons in turn requires simultaneous RNA- and DNA-based evidence to demonstrate the insertion of extraneous sequence in mRNA and distinguish transcriptional post-modification errors from deep intronic mutation in genomic DNA as the mechanistic basis of insertion. Hence, validated disease-associated pseudoexons have remained scarce [6, 20–22], despite the fact that potential pseudoexons are frequent in introns of human genes [18]. The pseudoexons in families 42 and 85 were missed by our original PTT screen [23]. Family 42 did reveal a visible truncation, but the subsequent search of a causative change by Sanger sequencing of genomic DNA did not extend deep into the introns [23]. On the other hand, no convincing extra fragment was visible for FAP85. This is likely attributable to some commonly observed disadvantages of PTT, such as decreased RNA stability and assay artifacts [24]. Instead of PTT, family 163 originally underwent an exon-by-exon screen in genomic DNA [8] that, obviously, was not able to capture deep intronic mutations.

Family 103 showed putative ASE imbalance (Table 1, Supplementary Table S2), but neither RNA-seq nor WGS revealed variants that might underlie the suggestive ASE phenotype. This apparently sporadic case with classical FAP (Figure 1) might be explained by a mosaicism for *APC* mutation; such mutations are challenging to detect and verify [5]. Eventual *in-cis* or *in-trans* regulatory changes or complex rearrangements escaping detection by sequencing would be examples of other theoretical possibilities to consider in future investigations. The ASE phenotype in FAP103 affected many other genes beyond *APC* (Supplementary Table S2), offering possible candidate genes to be tested for germline alterations. It is important to note that up to ~20% of all informative genes expressed in lymphoblastoid cells/blood may show ASE even in healthy control individuals [25, 26], and the underlying cause remains elusive for most genes.

The site of germline mutation in the *APC* gene is known to correlate with the disease phenotype [1]. Our family 42 with pseudoexon 6/7 was associated with classical FAP in agreement with genotype-phenotype expectations. Among the two pseudoexon 11/12 families, family 85 complied with established genotype-phenotype correlations by showing classical FAP like the two German families with the same mutation. Family 163 was classified as AFAP based on polyp count (10 – 20), but also showed features more typical (although not exclusive) of a profuse form of FAP such as low age at onset (16 years) and presence of extracolonic manifestations (mandibular osteomas) (Table 1). In FAP85, we demonstrated co-segregation of the respective genomic change with polyposis (Figure 1). Unfortunately, segregation studies were not possible in the remaining two families because of the lack of additional affected members.

Next-generation sequencing techniques are changing the screening for predisposing mutations. Targeted gene panels capturing the entire introns in addition to exons and combined with deep sequencing are likely to replace current screening protocols that rely on exon-specific Sanger sequencing and MLPA [4, 27]. We show that deep intronic mutations of the *APC* gene explained three out of four FAP and AFAP families displaying ASE imbalance and remaining mutation-negative by traditional methods. This indicates that our strategy to use ASE for pre-selection of cases for pseudoexon testing was effective and could even serve as a proxy for the initial screening of out-of-frame pseudoexon insertion events in FAP and AFAP. Unavailability of RNA made ASE and pseudoexon screening impossible in a significant fraction of our polyposis families (Table 1); hence, investigation of larger series is necessary for a reliable determination of the frequency and clinical significance of ASE and pseudoexon events in this disease. In the clinical context, pathogenicity of pseudoexons requires special attention. Considerations we point out (see Results above) as well as recommendations valid to any splicing aberrations [28] would apply. In our experience, next generation sequencing on RNA and genomic DNA facilitate pseudoexon identification and provide valuable tools to explore the genetic basis of mutation-negative families.

## MATERIALS AND METHODS

### Patients and samples

The series consisted of 54 unrelated families/cases from Finland, including 21 with classical FAP and 33 with AFAP (Table 1). Fourteen families represented a research-based cohort from the nation-wide Hereditary Colorectal Cancer Registry of Finland [13] lacking *APC* point mutations by PTT and exon-specific screening methods (heteroduplex analysis and Sanger sequencing) and large rearrangements by MLPA [8] (P043-C1). The remaining 40 families represented a clinic-based cohort of consecutive index cases with newly diagnosed FAP or AFAP and overlapped with the series described in ref. [8]. These cases were recruited via clinical genetic units of Finnish university hospitals, and cases remaining *APC* mutation-negative after exon-specific sequencing and MLPA were eligible (additionally, *APC* epimutations were excluded by methylation-specific multiplex ligation-dependent probe amplification [8]). *MUTYH*-positive cases and occasional cases with mutations in other polyposis-related genes were excluded. Cases with allele-specific expression (ASE) of *APC* were 42, 85-1, 85-2, 103, and 163 (ref. [13] and this study). No ASE was detected in cases 78, 88, 92, 97, 104, and 125 [13]. The remaining families/cases were uninformative or not tested for ASE because of the lack of RNA (as a rule, no RNA was available for clinic-based cases).

DNA and RNA were extracted from lymphocytes or EBV-transformed lymphoblasts as described [13]. This study was approved by the institutional review board of the Helsinki University Central Hospital (Helsinki, Finland).

### Single nucleotide primer extension (SNuPE)

SNuPE uses a single dideoxynucleotide (ddNTP) and a combination of three dNTPs for an extension reaction where the incorporation of a ddNTP yields differential extension of primers attached close to the polymorphic site [13]. Four coding single nucleotide polymorphisms (SNPs) in *APC* were used to study *APC* allele-specific expression (cDNA compared with gDNA) as described in Pavicic et al. [8]. ASE ratios (R) were validated against SNuPE results from individuals not carrying any *APC* mutation [8]. Ratios R≤0.6 or R≥1.67 were considered to indicate unequivocal ASE (40% reduction of one allele relative to the other allele) and 0.6<R<0.8 or 1.25<R<1.67 putative ASE (21 – 39% reduction of one allele relative to the other allele). The ASE statuses in Table 1 were assigned according to the highest ASE ratio yielded by any of the four coding polymorphisms.

### Transcriptome sequencing (RNA-seq) and transcriptome data-analysis

RNA-seq libraries were prepared using the ribo-depletion protocol from 12 DNAse treated total RNA samples, including three from FAP family 85 (individuals 85-1, 85-2, and 85-3), three from the index persons from families 42, 103 and 163, and three from proven mutation carriers from *APC-*mutation positive families 3, 93, and 63. RNA-seq data for three healthy individuals were generated for comparison. Sequencing of samples was done using Illumina HiSeq 2000 at the Institute for Molecular Medicine Finland (FIMM) (Helsinki, Finland). The bioinformatics workflow included correction of the sequence data for adapter sequences, bases with low quality, and reads less than 36-bp in length using Trimmomatics [29]. Paired-end reads passing the pre-processing were aligned to human reference genome build 38 (EnsEMBL v82) using STAR [30] with the default 2-pass multi-sample mapping settings, except that alignSJstitchMismatchNmax was set to 0 -1 -1 -1, outSJfilterCountUniqueMin to 6 2 2 2, outSJfilterCountTotalMin to 6 2 2 2, and outSJfilterDistToOtherSJmin to 10 0 0 0 in order to allow a more sensitive recovery of mutations at splice sites. Duplicate reads were marked with the Picard tools (http://picard.sourceforge.net) and strandedness information added with Bamutils [31]. Transcripts were assembled using StringTie [32] using the EnsEMBL v82 reference annotation file. Transcript predictions across all 12 samples were combined to a non-redundant set of transcripts using default parameters, except that minimum input transcript TPM and FPKM were set to 0.5.

### RNA-sequencing data variant calling and ASE analysis

Allele-specific expression of genes was quantified using Genome Analysis Toolkit (GATK) package [33] and ASE deceptions algorithm MBASED [34]. Briefly, pre-processed and mapped reads were split into exon segments using GATK SplitNCigarReads, local indel realignment was performed around indels using GATK IndelRealigner, and base qualities were recalibrated using GATK BaseQualityScoreRecalibration. Variants were called using GATK HaplotypeCaller and filtered using GATK VariantFiltration according to the best practice recommendations regarding the RNA-seq variant analysis workflow. Multi allelic sites were removed with GATK SelectVariants and non-heterozygous variants and variants falling outside of StringTie-called exon regions extended by 3 bp discareded with GATK VariantFiltration. The ASE deceptions algorithm MBASED [34] was then applied for each variant set to infer the probability of ASE in genes listed in EnsEMBL v82 and having ≥ 2 variants. Default non-phased ASE calling settings were used, except that dispersion estimate was set to 0.004 and the probability to detected haplotype 1 supporting reads was set to the average fraction of aligned reads supporting haplotype 1 variants with coverage ≥ 30 in the given sample. Sequence data was visualized using Integrative Genomics Viewer (IGV) browser [35]. Supplementary Table S3 outlines the performance of our RNA-seq and ASE experiments.

### Whole genome sequencing (WGS)

WGS was applied to DNAs from individuals 85-1, 85-2, and 85-3 from FAP family 85 as well as index patients from FAP families 42 and 103. Briefly, DNA was extracted from blood samples and KAPA and ThruPLEX sequencing libraries prepared according to the manufacturer's instructions. Sequencing was then conducted using Illumina HiSeq 2000 platform with KAPA and ThruPLEX libraries at the Institute for Molecular Medicine Finland (FIMM) (Helsinki, Finland). Sequencing data was analyzed by the FIMM variant calling pipeline version (VCP) 3.1 [36], including quality control of raw reads before and after alignment, pre-processing of reads for sequencing artifacts, alignment of reads to the human reference genome (build 19) using the Burrows-Wheeler Alignment (BWA) software [37], and calling of variants with the samtools package [38]. The minimum acceptable read depth for a variant was 7. Variant data were then analyzed by the VarSeq® software version 1.3.2 (Golden Helix, Inc., Bozeman, MT, www.goldenhelix.com). Genotype quality (difference between the Phred-scale likelihoods of the two most likely genotypes) was assessed on a scale between 0 and 99 and variants with genotype quality less than 70 excluded. All common variants with minor allele frequency (MAF) ≥0.001 were removed. Only heterozygote variants were considered in agreement with dominant inheritance in FAP family 85 and any inheritance pattern was accepted in the index patients from FAP families 42 and 103 (sporadic cases, Table 1). The identified variants were checked against ExAC (http://exac.broadinstitute.org) and SISu databases (www.sisuproject.fi) as well as Ensembl database (http://www.ensembl.org) to assess population frequencies. Sequence data was visualized using Integrative Genomics Viewer (IGV) browser [35]. Supplementary Table S4 lists some essential performance characteristics for the WGS experiments.

### Verfication of pseudoexons by sanger sequencing

To verify the pseudoexons identified by RNA-seq, relevant fragments of *APC* cDNA were amplified with primers from Spier et al. [6] and Sanger sequenced. The 11/12 pseudoexon was additionally verified from a cDNA fragment from exon 11 to exon 13 (amplified with primers given in Supplementary Table S1). Moreover, cDNA fragments 2 and exon 11 – 13 (Figure 3, Supplementary Table S1) were cloned into a pCR2.1 TOPO vector using the TOPO TA Cloning system (Invitrogen, Carlsbad, CA, USA) and DNAs extracted from the resulting white colonies were sequenced. The genomic variants discovered by WGS were confirmed by Sanger sequencing using primers around the respective nucleotide substitutions in introns 6 and 11 of *APC* (Supplementary Table S1).

### URL addresses for web resources used

Berkeley Drosophila Gene Project (BDGP), http://www.fruitfly.org/seq_tools/splice.html

Ensembl, http://www.ensembl.org

Exome Aggregation Consortium (ExAC), http://exac.broadinstitute.org

InSiGHT, http://insight-group.org

Sequencing Initiative Suomi (SISu), www.sisuproject.fi

PICARD, http://picard.sourceforge.net

GATK, https://software.broadinstitute.org/gatk/

VarSeq^TM^, http://www.goldenhelix.com

## SUPPLEMENTARY FIGURES AND TABLES




