# Supplementary material for: Pseudoexons provide a mechanism for allele-specific expression of APC in familial adenomatous polyposis
Source: Oncotarget. 2016 Sep 23;7(43):70685–98. doi: 10.18632/oncotarget.12206 (PMC5342583; doi:10.18632/oncotarget.12206)
Supplement: Supplementary file 1 [file oncotarget-07-70685-s001.pdf]

Pseudoexons provide a mechanism for allele-specific expression of *APC* in familial adenomatous polyposis

SUPPLEMENTARY FIGURES AND TABLES

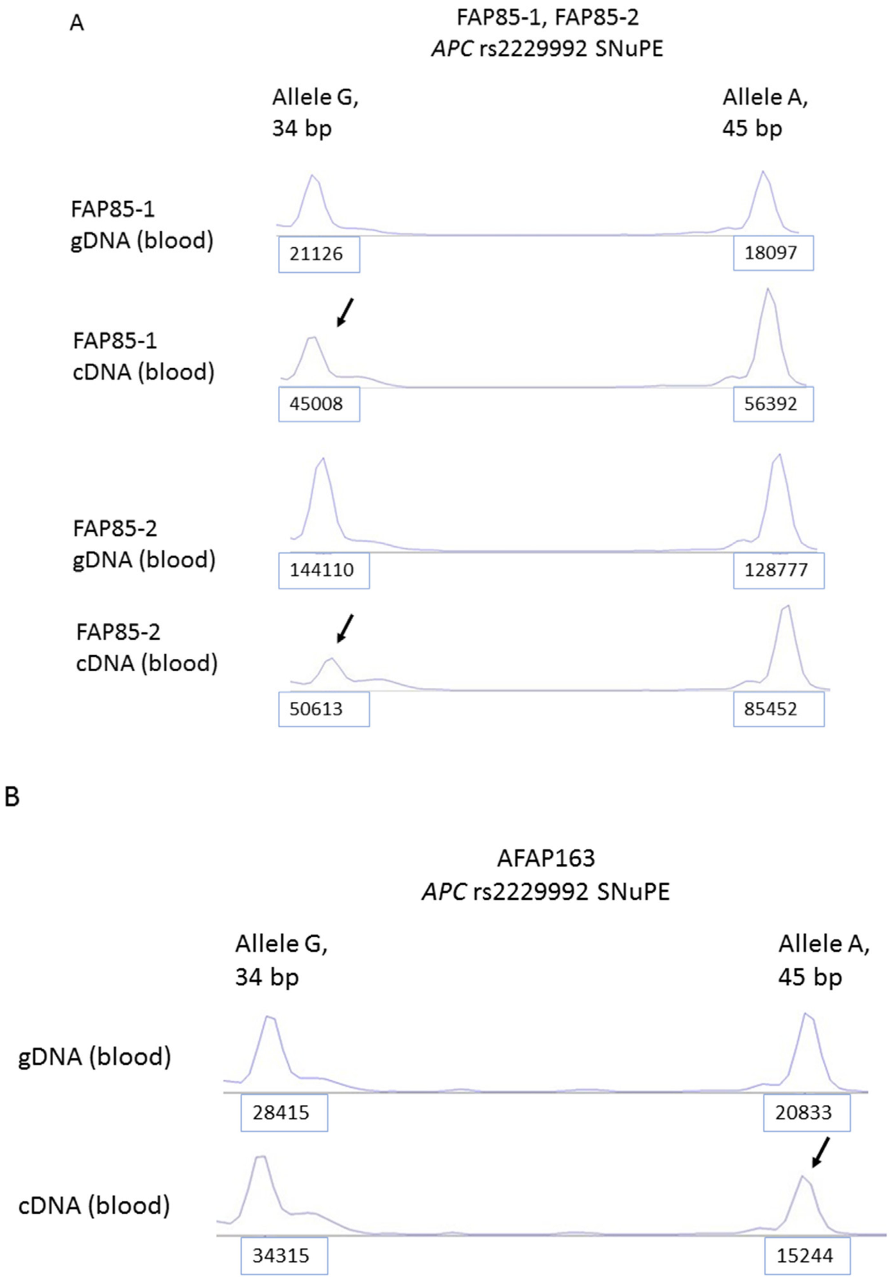

Supplementary Figure S1: SNuPE analysis to detect ASE of *APC*. The two *APC* alleles were distinguished based on a SNP from exon 12 (rs2229992) and their dosages assessed in blood-derived complementary DNA (cDNA) reverse transcribed from RNA, compared to genomic DNA (gDNA). Arrows denote the allele with reduced expression. The expression of allele G was decreased compared to allele A in individuals 85-1 and 85-2 from FAP85 (Supplementary Figure 1A) and that of allele A compared to allele G in AFAP163 (Supplementary Figure 1B). The peak ratios of G to A in cDNA relative to gDNA were 0.68 (85-1), 0.53 (85-2), and 1.7 (163) indicating ASE or putative ASE in all three samples (see Materials and Methods).

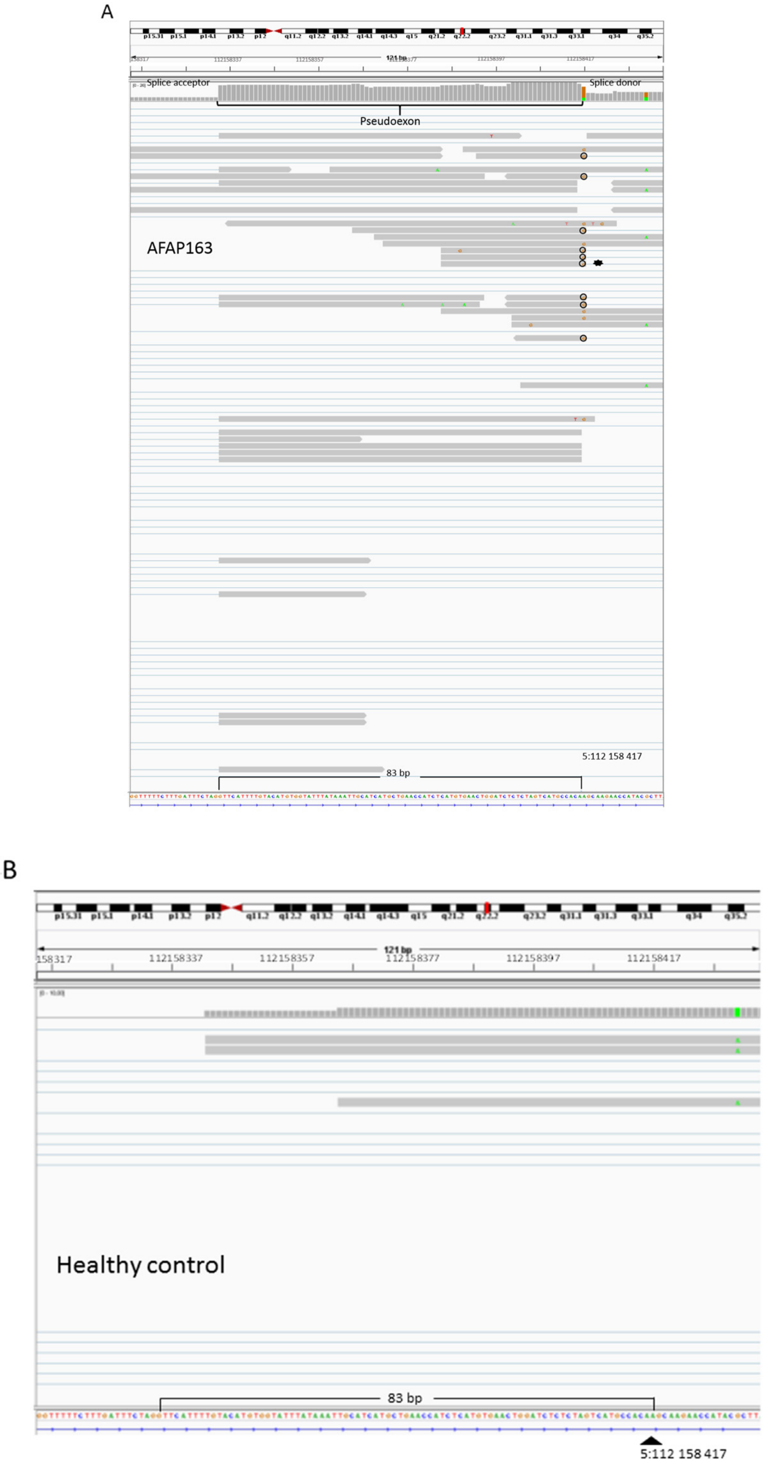

Supplementary Figure S2: A pile-up IGV image of individual reads from RNA-seq for the index person from AFAP163 (Supplementary Figure S1A) and healthy control (Supplementary Figure S2B). The figure provides a pile-up display of the same data that was given in a Sashimi plot format in Figure 2B. The borders of the 83-bp pseudoexon are indicated. AFAP163 shows 25 reads specific for the pseudoexon, 16 of which are associated with the splice acceptor and 9 associated with the splice donor. In the latter 9 reads, position 5:112158417, the site of the deep intronic mutation (Figure 5), constitutes the 3' end (circled). In all cases (9/9, 100 %), this position is occupied by the variant nucleotide (G). The result is in agreement with our cloning experiment for fragment ex11 – ex13 where all pseudoexon-containing cDNA clones (8/8, 100%) revealed G in this position. The asterisk denotes the single pseudoexon-specific transcript that was accompanied by a 54-bp insertion (see Figure 2B; the 54-bp insertion is located beyond the area of display of Supplementary Figure S2). No pseudoexon-specific RNA reads exist in a healthy control individual (Supplementary Figure S2B).

Supplementary Table S1: Primers for sequencing analysis of *APC* in cDNA and gDNA

Fragment designation	Template	Forward (5´)	Reverse (5´)	Length (bp)	Reference
Fragment 1	cDNA	TCTGTACCACCCTCAGTTCTG	GCAAGAAGCAATGACCTCTCT	575	Spier et al., 2012
Fragment 2	cDNA	TCTGGAGAGTGCAGTCCTGTT	CCTTGGTTCCCAGATGACTTG	615	Spier et al., 2012
Fragment 3	cDNA	GTGGGAGAAATCAACATGGCA	CCCATACATTTCACAGTCCAC	665	Spier et al., 2012
Fragment 4	cDNA	CAGATCTGTCCTGCTGTGTGT	AGTGCACCATCTACAGCACAT	505	Spier et al., 2012
Fragment 5	cDNA	GACGTTGCGAGAAGTTGGAAG	CAAGCTTGAGCCAGGAGACAT	564	Spier et al., 2012
APC exon 11-13	cDNA	CTGTCCTGCTGTGTGTGTTC	GCTCTCATGCAGCCTTTCAT	246	This study
APC intron 6	gDNA	GGCCAGAGTGGTCTCAATCTT	CCTAAATACAATGCCTTCATGCCT	200	This study
APC intron 11	gDNA	TGGTTTTTCTTTGATTTCTAGG	AACATAAACGGCACAAAAAG	171	This study

Supplementary Table S2: ASE results based on RNA-seq data (StringTie) vs. SNuPE.

See Supplementary File 1

Supplementary Table S3: Performance characteristics for RNA-seq and ASE analysis

	Read pairs (M)	Trimmed reads (M)	Properly mapped read (M)	Variants (total)	Accepted variants
FAP42	136,33	118,08	96,75	43050	18677
FAP85-1	81,92	71,36	64,76	40225	17678
FAP85-2	137,36	118,79	91,54	46675	20898
FAP85-3	142,75	124,73	96,22	42378	20569
AFAP163	110,07	90,53	86,51	123772	31517
FAP103	73,82	65,07	50,50	22786	11285
FAP3	96,38	82,44	73,13	10697	5444
FAP63	131,01	105,59	101,59	35225	18382
FAP93	75,38	67,70	65,10	12631	5862
control 1	116,78	93,12	88,06	34048	17229
control 2	102,88	70,22	67,63	26591	14291
control 3	115,45	94,38	90,50	138787	34587

Supplementary Table S4: Performance characteristics for WGS

FAP case	VCP-version	Genome size (bp)	Mean genome coverage	Number of reads before processing	Quality trimmed reads	Number of reads after PCR duplicates removed	Read length (bp)	Number of SNV variants	Number of indel variants
85-1	2,5	3095693981	16,2	745445432	97675851	12781568	93	3915997	419413
85-2	2,5	3095693981	13,7	612121114	79371793	10438636	93	4105808	404041
85-3	2,5	3095693981	7,2	345078096	44707929	4332537	93	3893147	312047
42	3,1	3095693981	8,1	373104720	57382420	7472734	125	3916556	324401
103	3,1	3095693981	7,2	341827692	57408228	6270309	125	3800895	308643
