# Supplementary material for: Pseudoexons provide a mechanism for allele-specific expression of APC in familial adenomatous polyposis
Source: Oncotarget. 2016 Sep 23;7(43):70685–98. doi: 10.18632/oncotarget.12206 (PMC5342583; doi:10.18632/oncotarget.12206)
Supplement: Supplementary file 2 [file oncotarget-07-70685-s002.docx]

| **Supplementary Table 2.** ASE results based on RNA-seq data (StringTie) vs. SNuPE. | | | | | | | | | |  |
| --- | --- | --- | --- | --- | --- | --- | --- | --- | --- | --- |
|  |  |  | |  |  |  |  |  | Genome-wide |  |
|  |  |  | |  |  |  |  |  | frequency of | *APC*-ASE by SNuPE |
| Case ID | EnsEMBL_ID | Gene Name | | Major Allele Frequency | p Value ASE | p Value Heterogeneity | q Value ASE^a^ | ASE_Is Significant^b^ | informative genes  with ASE (%) | (SNPs with ASE/all informative SNPs) |
| *Pseudoexon families* | | | |  |  |  |  |  |  |  |
| FAP42 | ENSG00000134982 | *APC* | | 0.736774080189033 | 0 | 0.165526 | 0 | 1 | 670/3597 (18.6%) | ASE (4/4) |
| FAP85-1 | ENSG00000134982 | *APC* | | 0.729908160509527 | 0.01641 | 0.318147 | 0.130830410958904 | 0 | 68/1582 (4.3%) | ASE (3/4), putative ASE (1/4) |
| FAP85-2 | ENSG00000134982 | *APC* | | 0.680950000027802 | 0.000113 | 0.155391 | 0.000624473684210527 | 1 | 1069/3905 (27.4%) | ASE (4/4) |
| FAP85-3 | ENSG00000134982 | *APC* | | NA | NA | NA | NA | NA | 1054/3975 (26.5%) | NI |
| AFAP163 | ENSG00000134982 | *APC* | | 0.587471456819093 | 0.131504 | 0.287946 | 0.186769423490326 | 0 | 620/3750 (16.5%) | Putative ASE (2/4), no ASE (2/4) |
| *No APC mutation found* | | | |  |  |  |  |  |  |  |
| FAP103 | ENSG00000134982 | *APC* | | 0.654445331391599 | 0.014144 | 0.684531 | 0.0612624351297405 | 0 | 282/2335 (12.1%) | Putative ASE (2/4), no ASE (2/4) |
| *Known APC mutation^c^* | | | |  |  |  |  |  |  |  |
| FAP3-1 | ENSG00000134982 | *APC* | | NA | NA | NA | NA | NA | 57/1064 (5.4%) | ASE (2/2) |
| FAP63-1 | ENSG00000134982 | *APC* | | 0.616946501942597 | 0.015026 | 0.198447 | 0.0187974661354582 | 1 | 1502/3788 (39.7%) | ASE (1/1) |
| FAP93 | ENSG00000134982 | *APC* | | NA | NA | NA | NA | NA | 70/1021 (6.9%) | ASE (4/4) |
| *Healthy controls* | |  | |  |  |  |  |  |  |  |
| Control 1 | ENSG00000134982 | *APC* | | NA | NA | NA | NA | NA | 567/3371 (16.8%) | NI |
| Control 2 | ENSG00000134982 | *APC* | | NA | NA | NA | NA | NA | 667/3022 (22.1%) | NI |
| Control 3 | ENSG00000134982 | *APC* | | 0.58933330659434 | 0.02426 | 0.004733 | 0.0545238856132076 | 0 | 801/3911 (20.5%) | Putative ASE (1/4), no ASE (3/4) |
| ^a^Corrected p-value | |  | |  |  |  |  |  |  |  |
| ^b^Statistically significant (1) or non-significant (0) at false discovery rate cut-off of 0.05.  ^c^The truncating mutations were c.839C>G (FAP3-1), c.471G>A (FAP63-1), and c.1548G>A (FAP93). | | | | | | | | | | |
| Abbreviations: NA, Not analyzed because of the lack of high-quality heterozygote variants; NI, Not informative (constitutionally homozygous) | | | | | | | | | | |
|  |  |  |  | |  |  |  |  |  |  |
